# Hospital Acquired Pneumonia Is Linked to Right Hemispheric Peri-Insular Stroke

**DOI:** 10.1371/journal.pone.0071141

**Published:** 2013-08-07

**Authors:** André Kemmling, Michael H. Lev, Seyedmehdi Payabvash, Rebecca A. Betensky, Jing Qian, Shihab Masrur, Lee H. Schwamm

**Affiliations:** 1 Division of Neuroradiology, Department of Radiology, Massachusetts General Hospital, Harvard Medical School, Boston, Massachusetts, United States of America; 2 Department of Biostatistics, Harvard School of Public Health, Boston, Massachusetts, United States of America; 3 Department of Diagnostic and Interventional Neuroradiology, University Medical Center Hamburg-Eppendorf, Hamburg, Germany; 4 Department of Neurology, Massachusetts General Hospital, Harvard Medical School, Boston, Massachusetts, United States of America; 5 Division of Biostatistics and Epidemiology, University of Massachusetts, Amherst, Massachusetts, United States of America; University of Münster, Germany

## Abstract

**Purpose:**

Hospital acquired pneumonia (HAP) is a major complication of stroke. We sought to determine associations between infarction of specific brain regions and HAP.

**Methods:**

215 consecutive acute stroke patients with HAP (2003–2009) were carefully matched with 215 non-pneumonia controls by gender, then NIHSS, then age. Admission imaging and binary masks of infarction were registered to MNI-152 space. Regional atlas and voxel-based log-odds were calculated to assess the relationship between infarct location and the likelihood of HAP. An independently validated penalized conditional logistic regression model was used to identify HAP associated imaging regions.

**Results:**

The HAP and control patients were well matched by gender (100%), age (95% within 5-years), NIHSS (98% within 1-point), infarct size, dysphagia, and six other clinical variables. Right hemispheric infarcts were more frequent in patients with HAP versus controls (43.3% vs. 34.0%, p = 0.054), whereas left hemispheric infarcts were more frequent in controls (56.7% vs. 44.7%, p = 0.012); there was no significant difference between groups in the rate of brainstem strokes (p = 1.0). Of the 10 most infarcted regions, only right insular cortex volume was different in HAP versus controls (20 vs. 12 ml, p = 0.02). In univariate analyses, the highest log-odds regions for pneumonia were right hemisphere, cerebellum, and brainstem. The best performing multivariate model selected 7 brain regions of infarction and 2 infarct volume-based variables independently associated with HAP.

**Conclusions:**

HAP is associated with right hemispheric peri-insular stroke. These associations may be related to autonomic modulation of immune mechanisms, supporting recent hypotheses of stroke mediated immune suppression.

## Introduction

Hospital acquired pneumonia (HAP) is a frequent sequelae of stroke and associated with higher mortality, worse neurological deficits, longer hospitalization, and increased costs for medical care [1]–[4]. At 7 days after stoke onset, HAP is the predominant medical complication in up to 25% of intensive care stroke patients [5]–[8]. The main general predictors for HAP after stroke include advanced age, gender and stroke severity [9]–[11]. Traditionally, HAP has been directly attributed to mechanical ventilation, aspiration from dysphagia and impaired cough reflex, immobility, and expiratory muscle weakness [2]. There is emerging evidence that brain infarction itself is a significant risk factor for infection due to brain–immune interactions after stroke [12]. Relating specific brain regions to post stroke infections requires robust statistical methods to curb false associations due to multiple comparisons, confounders and secondary effects. We sought to determine the relationship between infarction of specific brain regions and the risk of developing HAP using a matched case-control designed study in a validated penalized conditional logistic regression approach.

## Methods

### Study Population

A retrospective matched cases-control study was conducted in acute stroke patients developing HAP matched with non-pneumonia controls. This retrospective study was approved by the Institutional Review Board (Partners Human Research Committee) for research limited to the use of health/medical records under written consent and compliant with the Health Insurance Portability and Accountability Act. A total of 1977 acute ischemic stroke patients admitted to MGH stroke service within a four-year period (June 2004– March 2008) were screened consecutively and included for matching. Exclusion criteria were intracranial hemorrhage, infarcts in multiple vascular territories, signs of prior territorial infarct, any mention of pneumonia up to 48 h after admission, and CT/MR images that were unanalyzable. HAP was classified according to coding instructions of the Get With the Guidelines (GWTG)–Stroke program set forth in the CDC initiative of the Paul Coverdell National Acute Stroke Registry. Hereby, classification of HAP is contingent upon suspicion or mention of pneumonia in the medical record 48 hours or more after admission requiring antibiotic treatment. Patients without HAP defining criteria were categorized as controls. HAP patients were matched to controls by gender, NIH stroke scale score (NIHSS), and age, in descending order of priority.

Clinical data were obtained from each patient at baseline (age, gender, admission NIHSS, thrombolytic treatment, dysphagia as defined by MGH Swallow Screening Test, dyslipidemia, smoking history, coronary artery disease, diabetes mellitus, atrial fibrillation, hypertension) and discharge (in-hospital mortality, length of stay).

### Image Acquisition and Analysis

#### Imaging protocol

All CT scans were performed on a 64-detector row volume CT scanner (Light Speed; GE Medical Systems). MRI scans were performed using a 1.5 Tesla Signa whole body scanner (GE Medical Systems) with echo planar capabilities. Subacute ischemic brain lesions were outlined slice-by-slice in MRI-DWI/ADC (if available) or CT images. All images were chosen with an acquisition time closest to 48 h after onset of symptoms approximating final infarct size prior to infection. MR-DWI or CT images and respective binary lesion masks were affine registered to standard MNI-152 space (FLIRT 5.5, FMRIB Software Library) and manually corrected for registration errors (Analyze 11.0, AnalyzeDirect).

#### Voxel and region-based analyses

The primary infarct location (supratentorial right or left hemisphere, cerebellum, brainstem), distribution with regard to tissue type (gray- or white matter), infarct volume, and time of imaging were recorded. Percentage of infarction of distinct anatomic atlas based regions was determined.

First, lesion masks in MNI-152 space were segmented into 68 paired (left-right) brain regions (48 areas of the Harvard-Oxford [HO] cortical atlas; 20 subcortical/brainstem white matter areas of the Johns Hopkins University [JHU] white-matter atlas; atlases supplied by FMRIB Software Library). The atlases used were created by standardized anatomic labeling of multiple subjects linearly registered to MNI-152 standard space [13], [14], and the same type of registration method and tool was used in our study to reduce systematic errors introduced by differing registration methods. We used binarized atlases defining a specific structure with at least 25% probability of anatomic localization.

Percentage of infarction of supratentorial atlas brain regions was defined as the percentage of voxels of a region within a segmented infarct. Infratentorial areas were included in voxel-based analyses, but included in the atlas-based regional analyses only to the extent they are recognized white matter tracts (medial lemniscus, cerebellar and cerebral peduncles) because of limitation of reliability registering distinct small brainstem regions to standard space.

### Statistical analysis

Data are expressed as frequency (percentage) or mean ± standard error of the mean (SEM) or median (interquartile range). Patients’ clinical characteristics were compared using McNemar, Wilcoxon signed-rank tests, wherever appropriate.

A matched case-control study design was employed to identify infarcted brain regions significantly associated with HAP. Voxel-based and atlas-based regional-odds for HAP when infarction is present were determined. The log of the odds ratio (log-OR) at any voxel was calculated as a discordant pair analysis of voxels with vs. without infarction among HAP patients vs. matched controls. A voxelwise McNemar’s chi-square statistic was used to identify voxels that were significant (threshold p<0.05) in their association with HAP, along with the false discovery rate (q value) to estimate the expected proportion of false positives with multiple comparisons (FDR1.2, FMRIB Software Library) [15].

Risk of HAP by anatomical region was derived from atlas-based regional log-OR. The median percentage of infarction for each region was calculated across all included patients. A region was considered infarcted if its percentage of infarction was above the population median. For each region, the log-OR was calculated using conditional logistic regression with strata defined by the matched pairs. Region-based and voxel based log-odds ratio maps were displayed in MNI-152 space.

Using the matched case-control data we fit penalized conditional logistic regression models to identify imaging regions and clinical variables that are jointly significantly associated with pneumonia. We evaluated several approaches for inclusion of two-way interaction terms using 10-fold cross-validation. We then derived a prediction rule of developing HAP for future patients based on the selected imaging variables and the matching variables by leveraging the population from which the case-control study was sampled. Details of the statistical methods for variable selection with penalized regression approach and prediction are provided in the online supplement (Methods S1).

## Results

### Baseline Characteristics of Study Population

A total of 215 patients with HAP were matched with 215 controls obtaining an exact match by gender and high degree of matching by NIHSS (97.7% of the 215 pairs were within one point of each other, and the remaining 2.3% were within 4 points) and age (61.9% of the pairs were within 1 year of each other, 90.2% were within 3 years, 95.4% were within 5 years, 97.7% were within 7 years).

Average infarct volume was 13.9±1.7 ml in HAP patients compared to 13.4±2.0 ml in controls (p = 0.73). Baseline characteristics are listed in Table 1. There was no significant difference between the two study groups with regard to clinical characteristics, including dysphagia rate, except for longer hospitalization of HAP. The prevalence of traditional stroke risk factors was consistent with those reported in national registries [16].

**Table 1:** Clinical characteristics and short-term outcomes of HAP patients and matched controls.

	Pneumonia (n = 215)	Control (n = 215)	p Value
Age (years)	72.2±14.9	72.3±13.9	–
Male	116 (54%)	116 (54%)	–
Admission NIHSS	13 (6–19)	13 (6–19)	–
Dysphagia	151 (70.2%)	151 (70.2%)	1.00
Hypertension	146 (68.0%)	139 (64.7%)	0.53
Dyslipidemia	74 (34.4%)	68 (31.6%)	0.59
Diabetes mellitus	51 (23.7%)	44 (20.5%)	0.49
Atrial fibrillation	65 (30.2%)	58 (27.0%)	0.51
Smoking history	38 (17.7%)	35 (16.3%)	0.78
Coronary artery disease	59 (27.4%)	51 (23.7%)	0.39
Mortality	41 (19.1%)	38 (17.7%)	0.80
Length of hospitalization (days)	12.8±10.2	6.1±4.6	**<0.0001**

Age, Male and admission NIHSS were a priori matched by study design (see Results). Length of hospitalization by Wilcoxon signed-rank test and all other binary variables by McNemar test to account for the correlation caused by matching.

### Image and Statistical Analyses

There were no differences between controls and HAP patients with respect to median time between admission and follow-up imaging used for infarct segmentation (1.0 vs. 1.1 d; p = 0.57). The predominant infarct locations for the entire study population in descending order were the left hemisphere (n = 218), right hemisphere (n = 166), cerebellum (n = 33) and brainstem (n = 13). Left hemispheric infarcts were more frequent in controls vs. patients with HAP (56.7% vs. 44.7%, p = 0.012) whereas right hemispheric infarcts were more frequent in HAP vs. controls (43.3% vs. 34.0%, p = 0.054). The absolute number of brainstem lesions or tissue specific distribution of lesions did not differ between the two groups (Table 2).

**Table 2:** Predominant infarct location in HAP patients and controls.

	Pneumonia (n = 215)	Control (n = 215)	p Value†
Left hemisphere	96 (44.7%)	122 (56.7%)	**0.01**
Right hemisphere	93 (43.3%)	73 (34.0%)	**0.05**
Cerebellum	19 (8.8%)	14 (6.5%)	0.47
Brain stem	7 (3.3%)	6 (2.8%)	1.00
Gray matter*	127 (59.1%)	122 (56.7%)	0.68
White matter*	62 (28.8%)	73 (34.0%)	0.28

† based on McNemar test.

* supratentorial gray−/white matter excluding basal ganglia.

In univariate regional atlas-based analyses, we calculated the percentage of infarcted tissue in 138 brain regions. Table 3 displays the ten regions with the largest percentage of infarction, shown for all cases and broken down as HAP-patients vs. controls. In univariate analysis, left peri-sylvian regions were infarcted most often in the entire sample, whereas the right insular cortex was the only region significantly more frequently infarcted in HAP patients vs. controls.

**Table 3:** Brain regions most affected by infarction in HAP patients versus controls, displayed in descending order by mean percent infarction.

Brain region	All Patients	Pneumonia	Control	p Value
L External capsule	18.67±33.18	18.57±32.98	18.77±33.46	0.95
L Insular Cortex	18.52±33.97	18.35±34.12	18.69±33.90	0.92
L Central Opercular Cortex	18.49±34.60	18.64±34.90	18.33±34.38	0.93
L Superior fronto-occipital fasciculus	17.89±35.55	18.56±36.07	17.21±35.09	0.69
L Frontal Operculum Cortex	16.98±34.49	16.51±34.99	17.46±34.04	0.78
L Parietal Operculum Cortex	16.61±34.40	15.51±33.49	17.70±35.33	0.51
R Insular Cortex	16.12±32.06	19.72±34.82	12.52±28.68	**0.02**
L Superior longitudinal fasciculus	15.90±30.55	14.83±28.39	16.98±32.61	0.47
L Planum Temporale	15.65±32.48	13.86±30.88	17.44±33.98	0.25
L Heschl's Gyrus	14.44±33.56	15.90±32.35	17.92±34.78	0.53

L = left, R = right.

**Figure 1:**
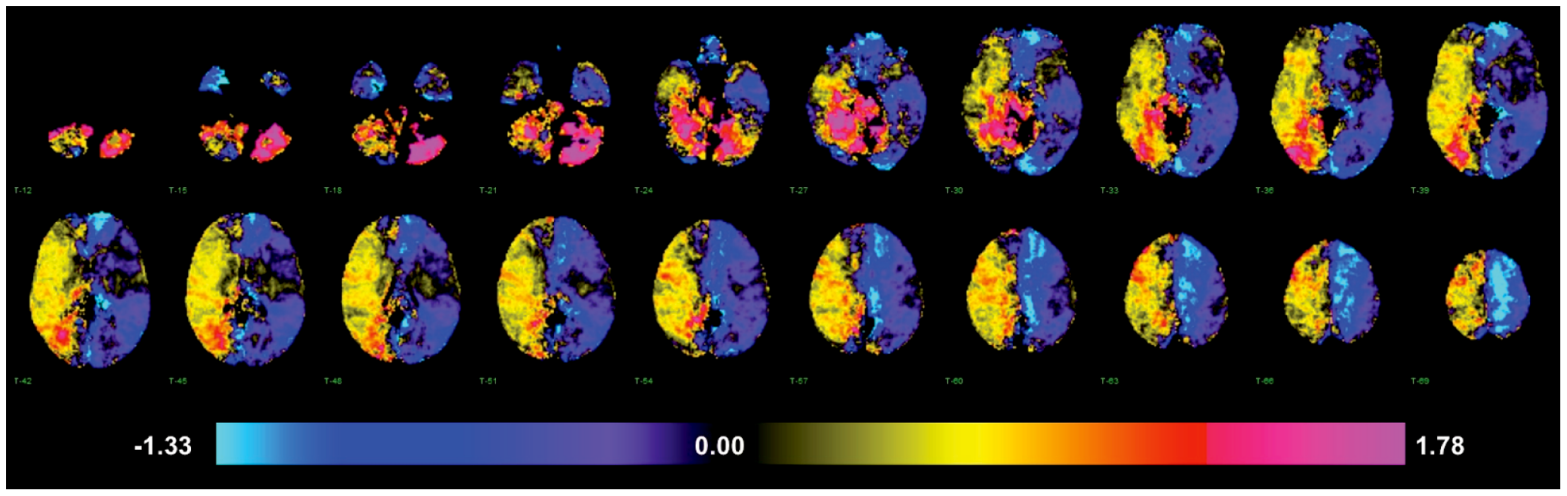
Voxel-wise odds for developing HAP when infarction present, expressed as log-OR (range −1.33 to 1.78; p<0.05).

**Figure 2:**
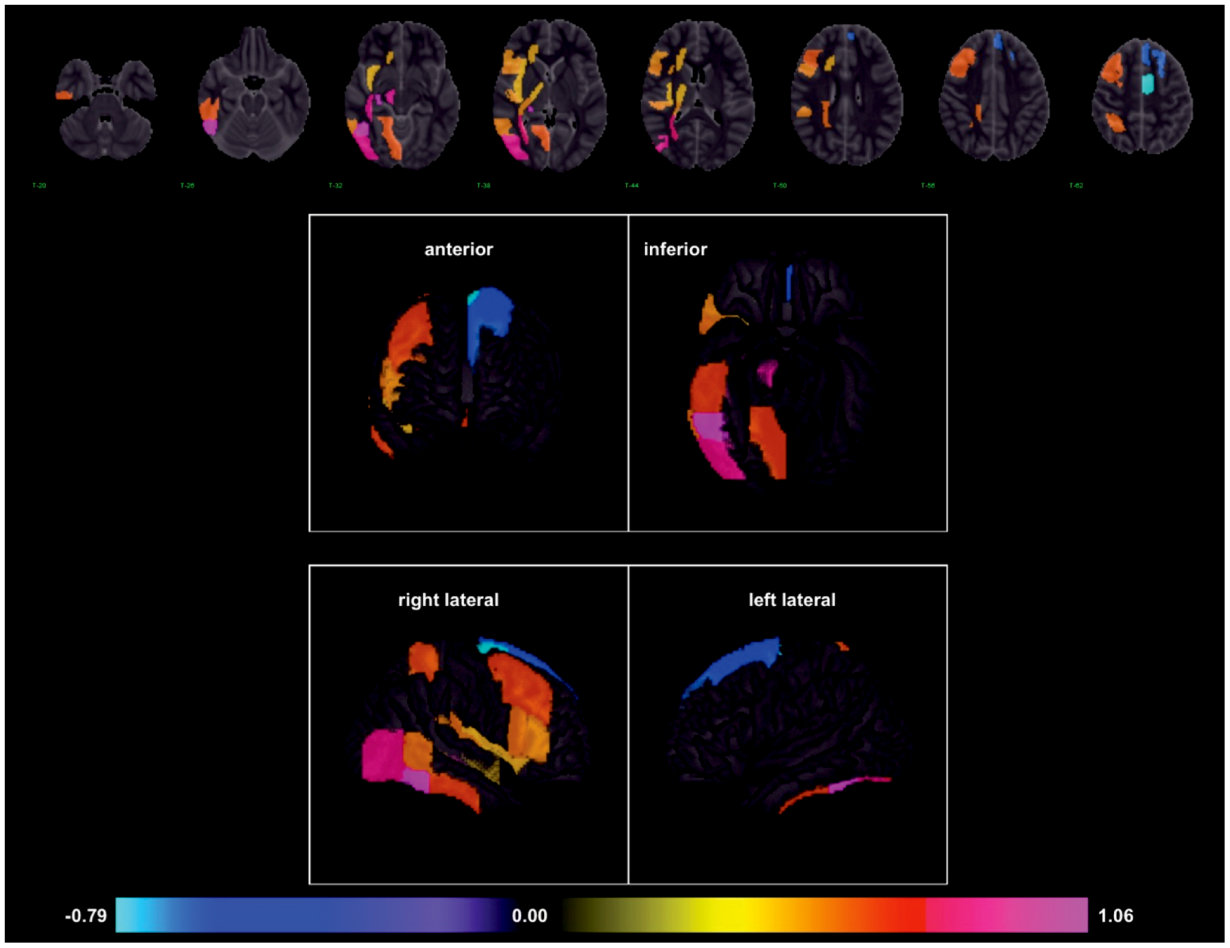
Infarcted brain regions with significant odds of developing HAP in stroke patients, expressed as log-OR (range −0.79 to 1.07; p<0.02). Displayed as axial and cortical 3-dimensional representations.

We performed univariate log-OR analyses to assess the relationship between individual areas of infarction and the likelihood of developing HAP. This was done at both the voxel level and at the level of atlas-based anatomic structures. In univariate voxel analyses, the highest log-ORs for pneumonia were observed for infarcted voxels located in the cerebellum, brainstem and right hemisphere, with the lowest observed in the left hemisphere (Figure 1). In atlas region-based analysis, these univariate relationships remained consistent with elevated log-OR for HAP in regions of the right hemisphere. Table 4 lists the univariate log-ORs for supratentorial regions potentially associated with HAP as defined by p<0.05 and their q-value to control for false associations that can emerge due to multiple testing [15]. A visual representation of the log-OR of listed regions with a threshold of significance set at p<0.02 is displayed in Figure 2, in axial and three dimensional reconstructions. Regional positive log-odds ratios for pneumonia were observed for infarcts in the right hemisphere. There were two left hemispheric regions with significant negative log-odds for pneumonia, suggesting they are associated with decreased odds of HAP.

**Table 4:** Univariate log-OR for supratentorial anatomic brain regions associated with HAP (as defined by p<0.05) and false discovery rate (q-value).

Brain region	log-OR	p Value	q Value
R Sagittal stratum	0.8544	0.0014	0.0415
R Fornix/Stria	1.0704	0.0014	0.0415
R temporooccipital InferiorTemporal Gyrus	1.0460	0.0012	0.0415
R Lateral Occipital Cortex	0.8557	0.0031	0.0708
R Posterior thalamic radiation	0.7691	0.0056	0.0865
R Middle Frontal Gyrus	0.6554	0.0057	0.0865
R Uncinate fasciculus	0.7178	0.0085	0.1099
R Posterior corona radiata	0.6286	0.0129	0.1168
L Superior Frontal Gyrus	**−**0.6286	0.0129	0.1168
L Juxtapositional Lobule Cortex	**−**0.7885	0.0113	0.1168
R Cerebral peduncle	0.8755	0.0200	0.1372
R retrolenticular Internal capsule	0.5653	0.0240	0.1372
R Inferior Frontal Gyrus	0.5831	0.0221	0.1372
R Superior Parietal Lobule	0.6391	0.0242	0.1372
R Frontal Operculum Cortex	0.5543	0.0217	0.1372
R Parietal Operculum Cortex	0.5831	0.0221	0.1372
R Heschl’s Gyrus	0.5596	0.0287	0.1533
R Insular Cortex	0.4855	0.0307	0.1546
R Inferior Frontal Gyrus	0.5534	0.0345	0.1644
R posterior Inferior Temporal Gyrus	0.6592	0.0382	0.1648
R Lingual Gyrus	0.6592	0.0382	0.1648
R Anterior corona radiata	0.5031	0.0429	0.1689
R Middle Temporal Gyrus	0.5819	0.0422	0.1689
R posterior limb Internal capsule	0.4884	0.0458	0.1697

L = left, R = right.

We identified imaging variables independently associated with risk of developing HAP in a multivariate conditional logistic regression model (Table A in Results S1). The best performing cross validated conditional logistic regression model (elastic net version of Pen3 in Methods S1) selected four regions of the HO cortical structural atlas (left superior frontal gyrus, right middle frontal gyrus, right inferior temporal gyrus, left juxtapositional lobule cortex) and three regions of the JHU white-matter atlas (right cerebral peduncle, right sagittal stratum, right stria terminalis) with multiple two-way interaction terms. In addition, two categorical imaging variables for infarct volume were included. The model (Equation 1 in Methods S1) uses the imaging variables (vector *X*), the matching variables age, gender, NIHSS (vector *Z*) and the estimates of the coefficients 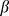 and 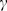 with intercept 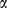 (Table B in Results S1) to calculate probability of the binary outcome variable HAP.

## Discussion

The aim of this study was to determine if the development of HAP after ischemic stroke was associated with infarction of specific brain regions when controlling for major clinical confounders in a matched case-control study design. A validated penalized conditional logistic regression approach was used to integrate both atlas-based brain regions and clinical patient characteristics while accounting for the interdependence of adjacent regions and correcting for multiple comparisons.

Patients who developed HAP had predominantly right hemispheric infarcts, compared to controls with a higher frequency of left sided lesions; the rate of dysphagia (Table 1) and brainstem strokes (Table 2) were similar between groups. A left hemispheric prevalence has been well documented among hospitalized stroke patients in general, due in part to the increased severity of deficits produced by left hemisphere strokes [17], [18]. Significant positive log-odds ratios suggest elevated risk for pneumonia for lesions located in the right hemisphere (particularly with involvement of insular, precentral, perisylvian and temporal cortex) except for two left hemispheric frontal regions with negative log-odds ratios (Table 4). The results suggest that strokes with HAP exhibit an asymmetrical distribution of infarct locations diametrical to strokes without HAP or a general mixed stroke population^16^.

Our study is consistent with prior evidence of lateralization dependent effects on neuro-immune responses [19]. A lateralized relationship between insular infarction and pneumonia may be postulated. The percentage of right insula infarction was significantly higher in HAP patients compared to controls. Additionally, odds for pneumonia were significantly elevated for infarcts within the right insula and peri-opercular cortex. This finding is in accordance with reports that implicate the right insular region in autonomically-induced immunosuppression and susceptibility to infection [20]–[22]. Similarly, right hemispheric peri-insular infarction attributes to autonomic dysfunction and pathologic sympathetic activity by diminishing cardiac vagal activity, relatively increasing sympathetic outflow and cardiac electrical irritability with adverse events [23].

Patients with right-sided brain lesions show significantly higher cutaneous T-lymphocyte reactivity on the paretic side [24], [25]. Specifically, an immunoregulatory lymphocytic role has been associated with the right frontal cortex [25], a region with significantly high odds for pneumonia in this study. Complementary, an independent association of the anterior middle cerebral artery cortex with post-stroke infections was reported recently [26].

The exact pathogenetic mechanisms with regard to a lateralized neuroimmune response as a risk factor for post stroke infections is still under debate and has been interpreted inconsistently. Low white blood- and T-cell counts from left hemispheric lesions have been interpreted as a risk factor for infections [27]–[29]. However, these studies only investigated an immunoregulatory response without recording the occurrence of infections by predetermined criteria and the results were not adjusted for relevant confounders such as patient stroke severity, age, or comorbidities.

Total infarct volume is a major predictor of infection [30]–[33]. For the outcome variable HAP, infarct volume was an independent variable with multiple interaction terms. This may relate to anatomic interconnectedness of location dependent effects on neural-immune modulation with a perturbed balance of excitatory and inhibitory signaling pathways [34].

Traditionally, post-stroke pneumonia is thought to develop mainly as a direct consequence of aspiration due to dysphagia and immobilization and up to 40% of patients with dysphagia are documented to aspirate. Brainstem strokes promote dysphagia related pneumonia particularly in concert with supratentorial per-insular lesions [6], [35]–[38]. However, although post-stroke aspiration increases the risk of pneumonia sevenfold, dysphagia alone is not sufficient to explain the high incidence of HAP when controlling for stroke severity [39]. This relates to the a posteriori observation in our study that there was no difference in the frequency of dysphagia or brainstem strokes between HAP patients and controls matched by main confounders.

In summary, the right hemispheric peri-insular cortex appears to be a site of vulnerability for developing HAP after stroke. Patients at higher risk may be identified, even if they do not fit the traditional clinical profile of those most at risk for aspiration. Our study suggests, that the increased attributable risk of right peri-insular infarction may be due more to impairments in host immunity than to the increased likelihood of aspiration. This observation may pose useful for initiating protective therapies targeting pharmacologic sympathetic blockade in patients at risk. Furthermore, there may be immunomodulatory infarction patterns that appear to be protective against HAP involving frontal left hemispheric regions associated with decreased odds for pneumonia. This observation should be further explored to identify potential therapeutic immunomodulatory targets.

Regional effects as presented may not be exclusively linked to pneumonia. Further stroke related infections, such as urinary tract infections, may share a common regulatory pathomechanisms imposed by a similar infarction pattern particularly with respect to right peri-insular lesions. However, pattern analysis with a deeper stratification by type of infection has been inconclusive in previous reports and will likely require a large-scale population study [31].

Our results are supported by a rigorous statistical method to identify true associations between brain regions and specific clinical outcomes. The cross-validated linear regression model may be useful for future investigations and stroke related risk assessment of HAP. Limitations arise from sample size and retrospective design. The matched case-control design was effective in controlling for critical risk variables but may have introduced inadvertent bias. Additionally, there may be unmeasured confounders that cannot be addressed without a larger sample or randomized prospective design. A further disadvantage is that conclusions with regard to prevalence and predictive value in a general inpatient stroke population are limited. Atlas-based analyses may have introduced systematic confounding by inherent technical limitations.

### Conclusion

Hospital acquired pneumonia is linked to right hemispheric peri-insular stroke. Our current study supports a hypothesis of secondary immunosuppression specific to lesion location.

## Supporting Information

Methods S1 Details of variable selection with penalized regression approach and prediction.
(DOCX)

Results S1 Multivariate logistic regression model for prediction of HAP.
(DOCX)
