# Supplementary material for: Hospital Acquired Pneumonia Is Linked to Right Hemispheric Peri-Insular Stroke
Source: PLoS One. 2013 Aug 7;8(8):e71141. doi: 10.1371/journal.pone.0071141 (PMC3737185; doi:10.1371/journal.pone.0071141)
Supplement: Methods S1 — Details of variable selection with penalized regression approach and prediction. (DOCX) [file pone.0071141.s001.docx]

**ONLINE SUPPLEMENT**

**METHODS S1 -** Details of variable selection with penalized regression approach and prediction

**Variable Selection**

We assumed a logistic regression model for the binary variable, HAP, on the population level,

$\mathrm{logit}[\Pr(HAP=1|X=x,Z=z)]=\alpha+\beta^{T}x+\gamma^{T}z$ (1)

where *X* is a vector containing the predictors (imaging variables) including two-way interactions and *Z* is a vector containing the matching variables (age, gender, NIHSS). Specific brain regions of infarction that are independently linked to HAP were determined by regression analysis.

We dichotomized the percentages of infarction for each imaging region at its median value. We constructed categorical variables for infarct volume using its tertiles (i.e., ≥ 33% and ≥ 67%). To make the analysis results more stable, we excluded imaging variables whose nonzero values were less than or equal to 5% in either cases or controls. There are no variables with fewer than 5% zero values. In total, we excluded 16 imaging variables due to their very low variation.

We considered several different variable selection approaches that allow for the very large set of predictors and that allow for interaction terms in the model. We compared the performances of stepwise regression, logic regression [[1](#_ENREF_1)], three L1 penalized regression methods [[2](#_ENREF_2)] named **Pen1** to **Pen3** and an “elastic net” penalized regression method [[3](#_ENREF_3)]. To accommodate the matched case-control design, we used conditional likelihood function in all the regression methods we compared. The stepwise regression starts from a model with no covariates, and uses combination of forward selection and backward elimination repeatedly with Akaike information criterion (AIC) as model selection criterion. Logic regression creates logic tree structure, where the leaves represent the binary predictors and the knots represent the Boolean expression of the original binary predictors. It is a useful tool to detect complicated interactions between binary predictors associated with a response variable when the interactions are of primary concern. By introducing “penalty functions” of regression coefficients into the likelihood, heuristically a cost for the number of parameters in the model, L1 penalized regression shrinks the regression coefficients toward zero and reduces the number of covariates with nonzero coefficients, and thereby implements variable selection. Given the fact that some imaging variables may be correlated, we also consider the elastic net method, which is a penalized regression with a mixture of L1 and L2 penalties. The model fitting and comparison was conducted in the R software [[4](#_ENREF_4)].

**Penalized Regression Approach**

The three approaches that we took for the penalized regression approach are:

**Pen1:** L1 penalized conditional logistic regression with main effects only.

**Pen2**:

- Stage 1: fit univariate conditional logistic regressions with each potential main effect and obtain a vector of p-values. With a pre-selected threshold for the p-values, we select the main effects for entry to the second stage.
- Stage 2: fit L1 penalized conditional logistic regression models with selected main effects from stage 1 and all of their two-way interaction effects; impose the penalty only on the two-way interaction effects.

**Pen3**:

- Stage 1: fit L1 penalized conditional logistic regression model with main effects only.
- Stage 2: fit L1 penalized conditional logistic regression model with selected main effects and all of their two-way interaction effects in the model; impose the penalty only on the two-way interaction effects.

Elastic net: To simplify the comparison, we evaluated elastic net variable selection procedure only for the procedure that corresponds to the one that performs best among **Pen1** to **Pen3**.

We used cross-validation to evaluate the different variable selection methods. We randomly divided the original 215 pairs of case-control observations into 10 groups consisting of 21 or 22 pairs. Using nine of the groups as the training dataset, we fit a conditional logistic model with each of the methods described above. Then we applied the fitted regression model to the one omitted group (the “test” set) to obtain the validation score, which we took to be the conditional log-likelihood for all observations in that group. We repeated this 10 times so that each group was used as the “test” set once, and we obtained the final cross-validation score by adding together the conditional log-likelihood for each “test” set. This cross-validated likelihood is commonly used as a criterion for evaluating variable selection methods [[5-7](#_ENREF_5)]. The higher the score, the better the variable selection procedure [[8](#_ENREF_8)].

**Prediction**

Let V=1 indicate that a subject is sampled into the matched case-control study and V=0 otherwise. Under the one-to-one matched case-control design, it follows from equation (1) that the probability of HAP among those patients sampled for the case-control study is

$\mathrm{logit}[\Pr(HAP=1|X=x,Z=z,V=1)]=\alpha+\text{ }f(z)+\beta^{T}x+\gamma^{T}z$ (2)

where$f(z)=\log[\Pr(HAP=0|Z=z)]-\log[\Pr(HAP=1|Z=z)]$. We estimated$f(z)$ using the population of 1977 acute ischemic stroke patients admitted to MGH stroke service, details of which can be found in [8]. Given the estimate of $\beta$ from the penalized conditional logistic regression at variable selection stage, we then fit the unconditional logistic regression model (2) to the matched case-control data by inserting $\hat{f}(z)$ and$\hat{\beta}^{T}x$ as offsets. This gives us estimates of $\alpha$ and $\gamma$ on population level. The predicted probability of developing HAP for future patients will be .

**References**

1. Ruczinski I, Kooperberg C, Leblanc M (2003) Logic Regression. Journal of Computational and Graphical Statistics 12: 475-511.

2. Tibshirani R (1996) Regression shrinkage and selection via the lasso. Journal of the Royal Statistical Society, Series B 58: 267-288.

3. Zou H, Hastie T (2005) Regularization and variable selection via the elastic net. Journal of the Royal Statistical Society, Series B 67: 301-320.

4. R-Development-Core-Team (2010) R: A language and environment for statistical computing.: R Foundation for Statistical Computing.

5. Le Cessie S, van Houwelingen J (1992) Ridge estimators in logistic regression. Journal of the Royal Statistical Society, Series C 41: 191-201.

6. Smyth P (2000) Model selection for probabilistic clustering using cross-validated likelihood. Statistics and Computing 10: 63-72.

7. Verweij PJ, Van Houwelingen HC (1994) Penalized likelihood in Cox regression. Statistics in medicine 13: 2427-2436.

8. Qian J, Payabvash S, Kemmling A, Lev MH, Schwamm LH, et al. (2013) Variable selection and prediction using a nested, matched case-control study: Application to hospital acquired pneumonia in stroke patients. Manuscript under review.
