# Supplementary material for: Hospital Acquired Pneumonia Is Linked to Right Hemispheric Peri-Insular Stroke
Source: PLoS One. 2013 Aug 7;8(8):e71141. doi: 10.1371/journal.pone.0071141 (PMC3737185; doi:10.1371/journal.pone.0071141)
Supplement: Results S1 — Multivariate logistic regression model for prediction of HAP. (DOCX) [file pone.0071141.s002.docx]

**ONLINE SUPPLEMENT**

**RESULTS S2** Multivariate logistic regression model for prediction of HAP

**Table A**

Atlas-based regions and infarction volume variables selected by penalized conditional logistic regression that are independently associated with risk of developing HAP.

| Label | Imaging variables independently associated with HAP |
| --- | --- |
| j10 | Cerebral peduncle R |
| j26 | Sagittal stratum R |
| j34 | Fornix (cres) / Stria terminalis R |
| h5 | Superior Frontal Gyrus L |
| h8 | Middle Frontal Gyrus R |
| h32 | Inferior Temporal Gyrus - temporooccipital part R |
| h51 | Juxtapositional Lobule Cortex L |
| VolBin1 | Volume greater than or equal to its 33% percentile |
| VolBin2 | Volume greater than or equal to its 67% percentile |
| 2-way Interactions | j10×h51, j10×Vol1, j10×Vol2, j26×h5, j26×h8, j26×h32, j26×Vol1, j34×h5, j34×h8, j34×h32, j34×Vol2, h5×h51, h5×Vol1, h8×h32, h8×h51, h8×Vol1, h8×Vol2, h32×Vol2, h51×Vol1 and h51×Vol2. |

h, Harvard-Oxford cortical structural atlas; j, Johns Hopkins University white-matter atlas; L, left side of brain; R, right side of brain

**Table B**

Estimates of regression coefficients for imaging predictors and matching variables.

| $x$ | J10 | J26 | J34 | H5 | H8 | H32 | H51 | VolBin1 |
| --- | --- | --- | --- | --- | --- | --- | --- | --- |
| $\hat{\beta}$ | -0.0701 | 0.4477 | 0.5684 | 1.4619 | 1.5089 | 3.1619 | 3.1834 | 0.4651 |
| $x$ | VolBin2 | J10 x H51 | J10 x VolBin1 | J10 x VolBin2 | J26 x H5 | J26 x H8 | J26 x H32 | J26 x VolBin1 |
| $\hat{\beta}$ | 0.9045 | -1.3149 | 1.9953 | -0.9385 | 1.3881 | -1.6748 | -3.9846 | 0.7036 |
| $x$ | J34 x H5 | J34 x H8 | J34 x H32 | J34 x VolBin2 | H5 x H51 | H5 x VolBin1 | H8 x H32 | H8 x H51 |
| $\hat{\beta}$ | 0.4764 | -0.5224 | -0.2467 | -0.0170 | -2.1236 | -2.5078 | 0.4950 | -0.5704 |
| $x$ | H8 x VolBin1 | H8 x VolBin2 | H32 x VolBin2 | H51 x VolBin1 | H51 x VolBin2 |  |  |  |
| $\hat{\beta}$ | -0.5420 | -0.3358 | -0.7411 | -1.4241 | -0.9733 |  |  |  |
| $z$ | Age | Sex | NIHSS |  |  |  |  |  |
| $\hat{\gamma}$ | 0.0049 | 0.1991 | 0.0837 |  |  |  |  |  |

**Note**: Binary variables: VolBin1 =$I(Volume\geq Q_{33}\left( \mathrm{Volume} \right))$, VolBin2 =$I(Volume\geq Q_{33}\left( \mathrm{Volume} \right))$, Sex (1=male, 0=female). Continuous integer variable: Age (years), NIHSS. Intercept $\alpha$ = -3.9381

The imaging variables listed in Table A are part of the multivariate model to predict HAP. Equation 1 in Methods S1 (online supplement) indicates how the values of these variables (summarized as vectors, *X* and *Z*) are combined (using the estimates of the coefficients $\beta$ and $\gamma$ with intercept $\alpha$ in Table S4) to calculate probability of HAP.
